# Health Benefits of Prebiotics, Probiotics, Synbiotics, and Postbiotics

**DOI:** 10.3390/nu16223955

**Published:** 2024-11-19

**Authors:** Nasser Al-Habsi, Maha Al-Khalili, Syed Ariful Haque, Moussa Elias, Nada Al Olqi, Tasnim Al Uraimi

**Affiliations:** 1 Department of Food Science and Nutrition, College of Agricultural and Marine Sciences, Sultan Qaboos University, Al-Khodh 123, Muscat P.O. Box 34, Oman; mahaalikh@gmail.com (M.A.-K.); moussa.habchy@gmail.com (M.E.); s89671@student.squ.edu.om (N.A.O.); s136129@student.squ.edu.om (T.A.U.); 2 Department of Marine Science and Fisheries, College of Agricultural and Marine Sciences, Sultan Qaboos University, Al-Khodh 123, Muscat P.O. Box 34, Oman; 3 Department of Fisheries, Bangamata Sheikh Fojilatunnesa Mujib Science and Technology University, Melandah, Jamalpur 2012, Bangladesh

**Keywords:** PPSPs, health benefits, gut microbiome, immune function, cholesterol-lowering effects, antimicrobial properties

## Abstract

The trillions of microbes that constitute the human gut microbiome play a crucial role in digestive health, immune response regulation, and psychological wellness. Maintaining gut microbiota is essential as metabolic diseases are associated with it. Functional food ingredients potentially improving gut health include prebiotics, probiotics, synbiotics, and postbiotics (PPSPs). While probiotics are living bacteria that provide health advantages when ingested sufficiently, prebiotics are non-digestible carbohydrates that support good gut bacteria. Synbiotics work together to improve immunity and intestinal health by combining probiotics and prebiotics. Postbiotics have also demonstrated numerous health advantages, such as bioactive molecules created during probiotic fermentation. According to a recent study, PPSPs can regulate the synthesis of metabolites, improve the integrity of the intestinal barrier, and change the gut microbiota composition to control metabolic illnesses. Additionally, the use of fecal microbiota transplantation (FMT) highlights the potential for restoring gut health through microbiota modulation, reinforcing the benefits of PPSPs in enhancing overall well-being. Research has shown that PPSPs provide several health benefits, such as improved immunological function, alleviation of symptoms associated with irritable bowel disease (IBD), decreased severity of allergies, and antibacterial and anti-inflammatory effects. Despite encouraging results, many unanswered questions remain about the scope of PPSPs’ health advantages. Extensive research is required to fully realize the potential of these functional food components in enhancing human health and well-being. Effective therapeutic and prophylactic measures require further investigation into the roles of PPSPs, specifically their immune-system-modulating, cholesterol-lowering, antioxidant, and anti-inflammatory characteristics.

## 1. Introduction

The human gut microbiome, a complex assemblage of trillions of microbes, has been recognized as a vital contributor to maintaining overall health and well-being. The gut response is crucial in maintaining optimal digestive function, regulating the immunological response, and impacting mental well-being [1]. Maintaining a delicate equilibrium in this microbial ecosystem is essential to ensure good physiological functioning.

The disruption of the gut microbiome has been associated with the emergence of many metabolic illnesses, highlighting the necessity for efficient approaches to foster and sustain this fragile microbial ecosystem. The functional dietary components referred to as prebiotics, probiotics, synbiotics, and postbiotics (PPSPs) have gained considerable attention in this setting [2].

Probiotics, by definition, are living organisms that must include a sufficient quantity of viable bacteria at the time of administration to the host [3,4]. Prebiotics are non-digestible fibers found in food that specifically support the growth and development of beneficial gut microorganisms [5,6,7], giving them the essential nutrients they need to flourish [1]. Probiotics are living bacteria that, when taken in sufficient quantities, can provide various health advantages to the host [1]. Synbiotics, which consist of prebiotics and probiotics [8,9,10], synergistically promote the survival and proliferation of beneficial gut bacteria, resulting in enhanced gastrointestinal health and general immunity [2]. The term “postbiotic” refers to the process of preparing inert bacteria and/or their components in a way that benefit the hosts’ health [11]. Postbiotics are bioactive substances that are created during the fermentation of probiotic bacteria [11,12,13,14,15]. These molecules have been associated with several beneficial health effects [1].

Recent findings indicate that PPSPs can manage metabolic disorders by influencing the makeup of the gut microbiome, improving the integrity of the intestinal barrier, and controlling the production of metabolites by the intestinal microbiota [1]. Research conducted both in laboratory settings (in vitro) and in living organisms (in vivo) has demonstrated that modifying the composition of gut microorganisms is a highly efficient method for treating metabolic illnesses using a type of therapeutic peptide that incorporates PPSPs [1].

Scientific research has extensively recorded various health advantages of these functional food constituents. These benefits include enhancing the immune system, relieving symptoms of irritable bowel disease (IBD), preventing and treating diarrhea, lessening the intensity of allergies, and demonstrating anti-inflammatory and antimicrobial characteristics [16]. Moreover, recent data indicate that PPSPs might enhance lipid profiles, reduce metabolic diseases, and improve overall immunological responses [1]. Although several probiotic strains have demonstrated encouraging signs of lowering cholesterol levels, conducting more comprehensive and extended randomized controlled trials is imperative to validate these effects on lipid profiles and cardiovascular well-being [17,18]. The immune-system-modulating ability of prebiotics must be validated over long periods, starting from early childhood [19,20].

Despite the growing interest and promising findings regarding prebiotics, probiotics, synbiotics, and postbiotics (PPSPs), substantial gaps remain in understanding their health benefits. This review aims to elucidate the significant advantages of PPSPs, particularly their antimicrobial, antioxidant, and anti-inflammatory properties, while also highlighting the transformative role of fecal microbiota transplantation (FMT) in addressing dysbiosis-related gastrointestinal disorders such as Clostridium difficile infection and Helicobacter pylori. By integrating insights from recent studies on PPSPs and FMT, this review advocates comprehensive research that combines nutritional therapy, preventive medicine, and sustainable practices to enhance gut health and optimize therapeutic strategies for metabolic diseases and other related health conditions.

## 2. Health Benefits

Prebiotics and probiotics enhance immune function by altering gut microbiota composition, resulting in elevated amounts of beneficial bacteria such as Bifidobacteria. They provide considerable cholesterol-lowering properties, diminishing total cholesterol, LDL cholesterol, and non-HDL cholesterol levels, thus enhancing cardiovascular health (Table 1). Postbiotics and synbiotics demonstrate anti-inflammatory effects, aiding in managing illnesses such as colitis and alleviating gastrointestinal discomfort. Furthermore, postbiotics and synbiotics have antioxidant activity, mitigating oxidative stress and promoting general health, especially in diabetes and metabolic disorders (Table 1). From the innumerable benefits of PPSPs, the key health benefits with roles, sources, and mechanisms of action are listed in Table 2.

### 2.1. Benefits of Prebiotics

Prebiotics are carbohydrates (fiber), that the human intestine cannot solubilize and digest. However, prebiotics are crucial in enhancing host cell functions [1]. Increasing evidence suggests that diets rich in prebiotics are linked to improved immune functions [43,44,45,46,47,48]. Soldi et al. [30] characterized the gut microbiota composition and explored the effects of the prebiotic intervention on the gut microbiota during a 24-week intervention and antibiotic treatment in healthy children. They observed the immune-system-modulating benefits while administering prebiotic inulin-type fructans, sometimes referred to as Orafti^®^, to healthy children [30]. The treatment was administered for 24 weeks and participants received 6 g/day of Orafti^®^. Results show improved immune functions due to increased levels of Bifidobacteria (*p* = 0.014) in the intervention group. The gut microbiota composition in children changed in response to prebiotic supplementation. This study shows that regular supplementation with inulin-type fructans can selectively modulate the gut microbiota [30]. Prebiotics may modulate the immune response by altering the composition of gut microbial populations or by generating microbial substances like short-chain fatty acids (SCFAs) [49,50,51,52]. In a study conducted by Cani et al., in 2009, prebiotics were administered to obese and diabetic mice with increased permeability accompanied by metabolic endotoxemia [53]. Intestinal barrier dysfunction or metabolic endotoxemia increases intestinal permeability, thus allowing inflammatory mediators from the gut to enter the bloodstream. As a result of prebiotics intake by diabetic and obese mice, the inflammation and metabolic symptoms were reduced. These symptoms include lower plasma lipopolysaccharide and cytokines and a decreased hepatic expression of inflammatory and oxidative stress markers [53]. In addition, prebiotic administration to the diabetic and obese mice group was accompanied by increased levels of specific tight-junction proteins (i.e., zona occludens 1 and occluding mRNA) in the Jejunum segment [53]. On the other hand, tight-junction proteins showed low levels in the tissues of the control mice group (i.e., fed a normal diet). These tight-junction proteins are known as key markers of tight-junction integrity [19]. This ultimately decreases inflammatory mediators’ ability to pass through the intestinal barrier. The increased endogenous glucagon-like peptide-2 (GLP-2) synthesis indicates that prebiotics enhanced gut barrier function via several potential mechanisms [53], mainly through an increased rate of crypt cell proliferation, villus elongation, and reduced apoptosis [54]. Additionally, GLP-2 administration increases intestinal insulin-like growth factor (IGF-I) secretion in vitro and enhances intestinal IGF-I mRNA transcript levels in vitro and in vivo [55]. Other supporting findings have shown that the GLP-2 receptor could be related to the activation of the b-catenin signaling pathway, which controls intestinal barrier function [56]. Prebiotics are bioactive components that can be used to treat and prevent metabolic disorders.

Prebiotic oligosaccharides have been used to alleviate atopic dermatitis (AD) and allergy symptoms in children under two years old [19]. In this instance, healthy children susceptible to atopy (genetic predisposition to allergic diseases) were introduced to one of the two following treatments: placebo hypoallergenic formula and prebiotic hypoallergenic formula. The formula was administered within the first six months of life, and results were evaluated during five years of life. The results show that introducing prebiotic oligosaccharides during the early stages of life could lower the progression of allergies in infants. The study did not extrapolate the exact mechanism of long-lasting prevention on allergy prevention/control by introducing the prebiotic oligosaccharides [19]. However, several explanations were addressed. Among these is that the modification of intestinal flora likely augments immunomodulation.

The composition of intestinal microbiota in early life strongly impacts the later occurrence of allergic cases. In addition, the dendritic cells of the intestinal mucosa play a significant role in the function of T cells (Treg), which are correlated to immune tolerance. Similarly, the administration of oligosaccharides showed improved symptoms in children suffering from atopic dermatitis (i.e., itching and aberrant epidermal lipid composition). These clinical improvements are associated with changes in the percentage of eosinophils in peripheral blood, which indicates that immunologic alteration contributes to the therapeutic effect of the prebiotics [57]. Nevertheless, the efficacy of prebiotic oligosaccharides against AD and allergic rhinoconjunctivitis still needs to be explored over the longer term [19,20]. The literature shows that prebiotic supplementation in children positively correlates with improved immune functions, as prebiotics modulate the gut microbiota selectively [49,58,59,60,61,62,63]. In addition, prebiotics have been associated with decreased inflammatory biomarkers in obese and diabetic mice [64]. This indicates that prebiotics could improve the host’s immune function. Some prebiotics directly affect the host’s immune system [43,49,52,65,66]. Wu et al. (2017) have reported that non-digestible OSCs modify host kinome and immune responses without altering gut microbiota and directly regulate host mucosal signaling, resulting in hyporesponsive intestinal epithelial cells to pathogen-induced protein kinase (MAPK) and nuclear factor kappa B (NF-_k_B). The kinase-phosphorylation events were mainly related to the potential mechanism of the immunomodulation of prebiotics. Nevertheless, posttranslational modifications could also be correlated to the ubiquitination, methylation, and SUMOylation that oversee host cell signal transduction regulation [66]. The mechanism of prebiotic action as an immune modulator could be related to either selective stimulation of bacteria growth (i.e., substrates provider) or direct immune stimulation [67].

### 2.2. Benefits of Probiotics

A growing number of studies have demonstrated that probiotics could provide cholesterol-lowering effects when administered in sufficient amounts [17,68]. For instance, scientists administered *Enterococcus faecium* CRL 183 and *Lactobacillus helveticus* 416 on 49 hypercholesterolemic healthy males to evaluate their effect on lowering cholesterol lipid profiles [22]. In this study, three groups of participants received different treatments: one set of participants received nothing except a soy product with probiotics (SP group); the second group received a soy product containing probiotics with isoflavones (ISP group), while the placebo group received unfermented soy products (USP). The results demonstrate a significant reduction in total cholesterol (by 13.8%), non-HDL-C (by 14.7%), and electronegative low-density lipoprotein cholesterol (LDL) (by 24%) among the ISP group. However, high-density lipoprotein cholesterol (HDL-C) levels remained the same. Groups receiving either SP or USP (placebo) treatment did not show a decrease in serum lipid profiles during the treatment period [22]. The hyperlipemia-relieving effect of probiotics in this study was attributed to several actions, including the assimilation of cholesterol, bile salts deconjugation, the production of short-chain fatty acids (SCFA) due to the fermentation of non-digestible carbohydrates, and microbiota modulation [69].

According to the study, probiotics significantly influence patients with high cholesterol [70,71,72,73]. The action occurs in the intestine tissues, where they can bind cholesterol, thus preventing its ingestion by the body. In addition, they play a role in the production of some biliary acids that act directly in lipid and cholesterol metabolism [74,75]. Some probiotics can produce SCFAs that prevent the formation of cholesterol in the liver [68,76]. Another study, conducted by Park et al., in 2018, investigated the cholesterol-lowering effects of *Lactobacillus rhamnosus* BFE5264 extracted from fermented milk [77]. The treatment results show a substantial decrease in cholesterol levels with *L. rhamnosus* BFE5264. Moreover, short-chain fatty acids (SCFA) were found to be produced in higher quantities in response to alterations in the gut microbiota. The mechanism of the cholesterol-lowering effect of BFE5264 was investigated by the analysis of the gut microbiota in cecal contents by the Illumine Miseq system. It was recognized that probiotic consumption could regulate the beneficial modulation of a dysbiotic-associated population (i.e., resulting from a high-fat diet) and consequently stabilize the intestinal microbiota [77]. Another study, conducted by Yoon et al., supported observation of a cholesterol-lowering effect on the part of BFE5264 treatment, where cholesterol efflux was significantly increased [67]. This increase was attributed to the upregulation of ABCG5/8 (i.e., primary neutral sterol transporter in hepatobiliary and transintestinal cholesterol excretion) through activation of liver X receptors (i.e., nuclear receptors that are activated by endogenous oxysterols, oxidized derivatives of cholesterol) [78].

In another instance, a randomized controlled trial investigated the cholesterol-lowering potential of yogurt formulations containing *Lactobacillus reuteri* NCIMB 30242 [21]. Subjects were hypercholesterolaemic males and females. The treatment was administered twice per day for a 6-week duration. Participants received randomized treatment (115 g yogurt + 10 g of microcapsule *L. reuteri* NCIMB 30242) or a placebo (125 g yogurt). According to the findings, *L. reuteri*, NCIMB 30242, was positively correlated with reduced lipid profiles among hypercholesterolaemic subjects [21]. The administration of *L. reuteri* NCIMB 30242 demonstrated significant reductions in LDL-C, TC, and non-HDL-C by 8.92, 4.81, 6.01%, respectively, compared with the control group [21]. In 2014, Guardamagna et al., investigated the effect of three probiotic strains, *B. animalis subspecies lactis* MB 2409, *B. bifidum* MB 109B, and B. longum subspecies longum BL04, which were lyophilized and formulated at a dosage of 1 × 10^9^ CFU each [79]. Children with dyslipidemia were administered the probiotic formulation containing three strains for one 1 month. The results indicate that the probiotic formulation reduced TC and LDL-C by 3.4 and 3.8%, respectively. Moreover, this study showed that combining dietary restriction and supplementation with Bifidobacterium probiotics can improve dyslipidemic children’s lipid profiles [79]. However, some species of bifidobacteria could effectively transform linoleic acid into conjugated linoleic acids, which are isomers known for their positive biological processes [80]. Conjugated linoleic acid synthesis is one way that bifidobacteria could influence probiotic properties. The production of conjugated linolenic acids from linoleic acid was hypothesized to be a detoxification mechanism adopted by bacterial cells [81].

The lowering effect observed in systemic endotoxin levels in the case of the probiotic group was attributed to the change in the gut microbiome due to the probiotic administration. The probiotic has a competitive inhibition nature with other bacterial components, as it adheres to the mucosa and the epithelial intestinal walls. Consequently, it reduces the endotoxin that is circulated and enhances the immune response in favor of the host [25,82]. In a study, Ecologic^®^ Barrier, a multi-strain probiotic, was evaluated for efficacy. The probiotic contained nine different strains (*B. bifidum* W23, *B. lactis* W51, *B. lactis* W52, *L. acidophilus* W37, *L. brevis* W63, *L. casei* W56, *L. salivarius* W24, *Lactococcus lactis* W19, and *Lactococcus lactis* W58) [66]. Participants were adults diagnosed with type 2 diabetes mellitus (T2DM); they administered a dosage of 2.5 × 10^9^ CFU/g twice per day, for a six-month duration. Significant reductions in endotoxin and glycemic parameters like glucose, insulin, and homeostasis model assessment for insulin resistance (HOMA-IR) were observed. Endotoxin, glucose, insulin, and HOMA-IR decreased by 70, 38, 38, and 64%, respectively. In addition, supplementation of probiotics reduced both total cholesterol (TC) and high-density lipoprotein (HDL) ratio (TC: HDL) by 19%. The results indicate that multi-strain probiotic supplementation may improve metabolic parameters associated with diabetes and cardiovascular diseases [83].

Probiotics have been suggested as a therapeutic supplement for type 2 diabetes mellitus patients to enhance dyslipidemia and promote better metabolic control. The immunoregulatory properties of probiotics form the potential mechanism of probiotic action toward the control of type 2 diabetes mellitus [84,85,86,87]. Sohn et al., in a 12-week double-blind, randomized controlled study, found that consuming a supplement containing *Lactobacillus plantarum* K50 (LPK)—an isolated strain of kimchi—had many positive effects on healthy persons with a body mass index (BMI) of 25 to 30 kg/m^2^ [78]. These include reduced triglyceride and cholesterol levels, modifications to obesity, and changes to the gut microbiota, characterized by a rise in *Lactobacillus plantarum* and a drop in *Actinobacteria* [88]. The reductions in lipid profile by LPK in this study were justified by the increases in short-chain fatty acids (SCFAs) and changes in the gut microbiota composition [88].

Additionally, butyrate and propionate (i.e., other types of SCFAs) showed a preventive effect against diet-induced obesity [89]. These SCFAs (butyrate and propionate) controlled gut hormones, including glucagon-like peptide-1, glucose-dependent insulinotropic polypeptide (GIP), peptide YY, and ghrelin. In particular, GIP adjusts TG turnover and promotes TG clearance from the blood by increasing adipocyte fat deposition [90]. In 2018, Abbasi et al., evaluated *Lactobacillus plantarum* A7 for its effect on lipid profiles in patients with nephropathy and type 2 diabetes mellitus (T2DM) [80]. Participants received 200 mL/day of soy milk and were administered a 2 × 10^9^ CFU daily for 8 weeks [91]. Results show reductions in the levels of LDL-C (by 9.2 ± 10.4), TC (by 12.4 ± 4.8), non-HDL-C (by 15.3 ± 4.5), and triglycerides (TG) (by 14.6 ± 12.5). However, levels of HDL-C (1.11 ± 3.38) and serum phosphorus (−0.14 ± 0.10) showed no significant change. These findings indicate that supplementing soy milk containing *L. plantarum* A7 could improve lipid profile and glomerular function in T2DM patients [91]. Overall, the results of previous studies prove that supplementing probiotics to diabetic, hypercholesterolaemic, and dyslipidemic individuals can help improve lipid profiles significantly. Most studies discussed in this review report decreased TC, LDL-C, and non-HDL-C levels compared with baseline. However, some studies do not report significant changes in lipid profiles upon consumption of probiotics [92]. These contradictory results call for the urgent need to conduct more long-term human studies. Studies on probiotic strains enhancing lipid profiles and metabolic indices show promise, but more validation is necessary to confirm consistency and long-term therapeutic effectiveness.

### 2.3. Benefits of Postbiotics and Synbiotics

Postbiotics and synbiotics are cutting-edge areas in nutritional science that provide significant health advantages due to their distinct modes of operation. Postbiotics result from probiotic bacterial fermentation and have been linked to improved gut health, immunological regulation, and potential protection against several diseases. Synbiotics, which include probiotics and prebiotics, work together to improve the survival and growth of good gut bacteria, leading to better gastrointestinal health and overall immunity. This highlights a comprehensive strategy for food supplementation to enhance health results by influencing the gut flora.

#### 2.3.1. Antimicrobial Properties

Postbiotics and synbiotics showed positive effects in reducing the number of disease-causing bacteria [93,94,95]. For example, an in vivo study conducted by Gupta et al., in 2021, showed that *Lactobacillus* spp. and its nutraceutical compounds reduce intestinal inflammation in mice by lowering the infectivity of *Escherichia coli* [32]. The mechanism of immunomodulation provoked by probiotic *Lactobacillus* spp. in the existence of *Escherichia coli* was justified by the suppression effect of *Lactobacillus* spp. against the expression of NF-κB in the case of *Escherichia coli* [84]. The regulated NF-κB-dependent signaling is crucial for efficient immune response; however, prolonged activation initiates the generation of inflammatory diseases [96]. In 2024, Cirat et al., demonstrated this mechanism by attributing the bactericidal activity of *Lactobacillus* strains to the synergistic action of lactic acid and secreted bacteriocins [97]. Moreover, a study has been conducted to determine the role of synbiotics on broilers’ intestinal flora and histomorphology [39]. Over six weeks, 25 birds were fed on a basic diet and synbiotics (*S. cerevisiae* + Mannan Oligosaccharides). The intestinal microbiota composition was assayed, and broilers supplemented with synbiotics had significantly higher final body weight (BW) and feed conversion effectiveness than the placebo group. The average body weight was determined in 42 days for the synbiotic group as 2299.19 g, and 2058.1 g for the placebo group. Synbiotic supplementation also helped decrease the number of pathogens, such as *Escherichia coli*, in the small intestine and cecum. On day 21, the total count of *E. coli* in the small intestine was 7.49 log CFU/g for the synbiotic group, while for the control group it was 9.79 log CFU/g. Even in the cecum, the total count of *E. coli* was 5.93 log CFU/g for the synbiotic group and 7.43 log CFU/g for the control [39].

Additionally, Pan et al., stated in 2022 that, after a thorough investigation, postbiotics derived from *Lactobacillus paracasei* CCFM1224 effectively prevent non-alcoholic fatty liver disease by influencing the composition of the gut microbiota and the metabolic processes in the liver [98]. The authors investigated the prophylactic properties of postbiotics derived from *Lactobacillus paracasei* on non-alcoholic fatty liver disease (NAFLD). The findings demonstrate that concurrent consumption of a high-fat diet (HFD) and postprandial oxidative stress (POST) in mice results in a deceleration of weight gain, suppression of hypertrophy in epididymal white fat and insulin resistance, improvement in the serum biochemical markers associated with blood lipid metabolism, and a reduction in hepatic steatosis and liver inflammation. Bacterial sequencing revealed that POST had an impact on the gut microbiota in mice fed a high-fat diet (HFD), increasing the proportion of Akkermansia and decreasing the proportion of *Lachnospiraceae NK4A136* group, *Ruminiclostridium*, and *Bilophila*. The findings indicate that POST has a beneficial effect in preventing NAFLD [88]. This effect is likely achieved by influencing gut microbiota and liver metabolism. These findings may have implications for the development of functional foods targeting NAFLD.

#### 2.3.2. Antioxidant Properties

Postbiotics and synbiotics have been shown to have an ability to improve antioxidant properties [1,2,99,100,101,102]. In an in vivo study, Izuddin et al., used *L. plantarum* strain dietary postbiotics for lambs and was able to report improved antioxidant activity in serum and rumen barrier function in the postbiotic group [33]. Additionally, nutritional postbiotics improved the antioxidant activity in serum, and ruminal fluid decreased the serum lipid peroxidation, while antioxidant enzymes in the liver increased rumen barrier function [33]. The potency of *Lactobacillus plantarum* as an antioxidant is mainly related to its resistance to hydrogen peroxide and its strong scavenging activity in opposition to hydroxyl, superoxide, and DDPH free radicals [103]. The antioxidant properties of *L. plantarum* can be considered in addition to an intracellular antioxidant enzyme system [104].

Another study was conducted by Mirmiranpour et al., in 2020 to investigate the effect of cinnamon powder and probiotics from *Lactobacillus acidophilus* and their interaction with glycemic indexes and antioxidant profiles in patients with T2DM [37]. Patients (136) with T2DM were randomly divided into four clusters based on age and gender. In addition to regular medication, the *Lactobacillus acidophilus* 10^8^ CFU and 0.5 g of cinnamon powder were given to one of these groups (synbiotic). The results show that, after three months of treatment, the average fasting blood sugar (FBS) levels significantly decreased from 177.3 ± 23.02 mg/dL to 147.70 ± 3.71 mg/dL in the synbiotics supplementation groups associated with the placebo group, and antioxidant enzyme activity increased from 3.99 ± 0.27 U/mL to 15.88 ± 1.98 mU/L in the synbiotics groups [37]. It was proposed that lactic acid accumulation in the epithelium of the intestine could reduce glucose absorption in the intestine [105]. Additionally, probiotics delay pancreatic degradation by reducing the production of pro-inflammatory cytokines [106,107]. The antimicrobial and antioxidant activities contributed to the cinnamon, which extracts an inhibitory effect against the tumors, cyclooxygenase 2, and nitric oxide synthase enzymes [108]. Furthermore, cinnamon contains the essential oil cinnamaldehyde which has antimicrobial, antioxidant, anti-inflammatory, and anti-spasmodic properties [37].

Similar positive results emerged from a study by Soleimani et al., where the intervention of *L. acidophilus*, *L. casei*, and *B. bifidum*, in combination with inulin (i.e., a synbiotics capsule), for 60 diabetic HD patients for 12 weeks, considerably improved health metrics [38]. It was observed that synbiotic supplementation significantly reduced fasting plasma glucose, insulin levels, and insulin resistance while increasing insulin sensitivity compared with the placebo. Furthermore, consuming synbiotics led to a notable decrease in high-sensitivity *C*-reactive protein levels (β −2930.48 ng/mL; 95% CI, −3741.15, −2119.80; *p* < 0.001) and malondialdehyde levels (β −0.60 μmol/L; 95% CI, −0.99, −0.20; *p* = 0.003). In addition, there was a notable rise in both total antioxidant capacity (β 142.99 mmol/L; 95% CI, 61.72, 224.25; *p* = 0.001) and total glutathione levels (β 131.11 μmol/L; 95% CI, 89.35, 172.87; *p* < 0.001) in the synbiotic group as compared with the placebo group. Overall. synbiotic treatment for 12 weeks improved glycemic control, inflammatory biomarkers, and oxidative stress in diabetic hemodialysis patients [38]. The ingestion of probiotics and synbiotics could reduce the development of pro-inflammatory cytokines, reduce lipid peroxidation, and enhance the activity of antioxidant enzymes [109]. There are substantial investments in sustainable antioxidant formulations, prioritizing ecological production and green extraction approaches to address oxidative disorders and uphold effective standards.

#### 2.3.3. Anti-Inflammatory Properties

Many studies have been conducted in recent decades on the potential health benefits of postbiotics and synbiotics as anti-inflammatory agents [110,111,112,113]. It has been shown that these dietary supplements, known as functional foods, can change, modify, and restore the pre-existing intestinal flora [114]. In 2021, Neyrinck et al., proved a positive effect of *Lactobacillus bulgaricus* and *S. thermophilus* strains on the digestive system of mice with the inflammatory disorder colitis [42]. Their results indicate reduced inflammation, as the postbiotics reduced the disease progression [42]. Additionally, a clinical study included 27 patients of T2DM administrated with *Bifidobacterium lactis* in combination with fructo-oligosaccharides daily for 30 days. The results of the study show reduced gastrointestinal discomfort while lowering plasma levels and interferon-gamma (IFNγ) [42]. The impact of inulin and a novel symbiotic on the development and functional state of the gastrointestinal canal of calves was studied by Jonova et al. (2021) [40]. They found that adding prebiotic inulin to calves’ feed raises the pH in the rumen, abomasum, and intestines [107]. However, when inulin was combined with *S. cerevisiae*, the pH decreased and showed a lower level than in the control group [40].

Prebiotic inulin and the synbiotic produced when it is combined with yeast *S. cerevisiae* had a good effect on developing several morphological features in the rumen and gut of calves. Calves in the synbiotic group displayed superior outcomes across all criteria. Another study sought to identify the impact of synbiotic supplementation on cardiovascular disease indicators in gestational diabetes mellitus (GDM) women. Cardiovascular diseases (CVDs) are more likely to develop in GDM women. However, it has been shown that synbiotics have positive effects on the atherogenic index of plasma (AIP), inflammatory markers, and oxidative stress markers, all of which are known to be cardiovascular disease risk factors [108]. The group of researchers investigated the effects of synbiotics on pregnant women. The pregnant women with gestational diabetes mellitus were divided into two groups [108]. One group received a synbiotic capsule daily containing [500 mg of *L. acidophilus* (5 × 10^10^ CFU/g), *L. plantarum* (1.5 × 10^10^ CFU/g), *L. fermentum* (7 × 10^9^ CFU/g), *L. Gasseri* (2 × 10^10^ CFU/g) and 38.5 mg of fructo-oligo-saccharides] and was compared with a control group for 6 weeks. The study’s main findings indicate that pregnant women who consume synbiotic supplements for six weeks have a lower logTG/HDL-C (0.43) proportion than those who take a placebo (0.55). A crucial atherogenic metric, log TG/HDL-C, significantly decreased, indicating that synbiotics may have a CVD-preventive impact in GDM-pregnant women who are more likely to experience future CVDs [36]. However, many studies are advised to validate the advantageous effects of synbiotics, which also requires many postbiotic clinical trials. Studies verify that postbiotic and synbiotic combinations provide anti-inflammatory characteristics that benefit gastrointestinal health. Nevertheless, thorough clinical trials and optimization are necessary to validate these findings and prevent chronic illnesses.

#### 2.3.4. Quality Properties of Food

A study has indicated that people who consume postbiotics and synbiotics show improved immune function, balanced gut microbiota, reduced diarrhea, higher amounts of lactobacilli and bifidobacteria, and decreased infections [9]. In contrast, another study has also illustrated their role in functional foods where the use of the postbiotic *Lactobacillus rhamnosus*, in combination with the exopolysaccharide in cheddar cheese, contributed to an increase in moisture content and improvement in cheese yield and texture, which improved the product’s characteristics [18]. Additionally, Dell et al., conducted an in vitro study that used a supernatant of *Lactobacillus sakei* as a postbiotic in grilled meat, showing positive results in lowering the number of pathogenic bacteria such as *Escherichia coli* and *Listeria monocytogenes* [35]. A double-blind, randomized controlled trial was conducted to investigate the effects of administering a supplement called Bimuno^®^ galactooligosaccharide (B-GOS^®^) to 30 autistic children [29]. Each participant received a dose of 1.8 g of the supplement containing 80% GOS. This supplementation was followed for six weeks. The findings improved the composition of the bacteria residing in the children’s gastrointestinal tract. More specifically, the levels *of Faecalibacterium prausnitzii* and *Bacteroides* spp. increased, while the levels of *Bifidobacterium* spp., Veillonellaceae family, and Lachnospiraceae family decreased. Patients reported reduced abdominal pain and a positive change in bowel motions [29].

Furthermore, a study was conducted by Huan et al., in 2020 to investigate the utilization of postbiotics in yoghurt and to evaluate their benefits [115]. The polysaccharide from *Lactarius volemus* (*Fr*.) was extracted for use as a growth factor when fully integrated into three various probiotic yogurts. The results of the study show that pH was reduced for *Lactobacillus casei* from 4.49 to 4.28, *Lactobacillus acidophilus* from 3.88 to 3.77, *Lactobacillus plantarum* from 3.94 to 3.77, and that water retention capacity was significantly improved from 99.2 to 99.8, 99.3 to 99.5, and 98.6 to 99.2 respectively. Therefore, the research reveals that *L. volemus Fr*. polysaccharide extracts are a highly effective growth factor for shortening the fermentation time and enhancing the quality of probiotics yogurt [115]. Similarly, utilizing *L. plantarum* supernatant as a postbiotic in soybeans positively extended the shelf life to 2 months, with less mold, moisture, and toxins produced [116]. The nutritional value was improved without adversely affecting the food product properties [116]. Increased flavor, texture, and yield are just a few possible advantages of postbiotic chemicals in food. However, cost–benefit analyses and randomized controlled trials with extensive follow-up periods are required before they become widespread. The best postbiotic food designs result from collaborative efforts amongst every relevant group.

## 3. Impact of Fecal Microbiota Transplantation

Fecal microbiota transplantation (FMT) has emerged as a significant therapeutic approach for various gastrointestinal disorders associated with dysbiosis, particularly in the context of *Clostridium difficile* infection (CDI) and *Helicobacter pylori* [117,118]. These conditions not only highlight the efficacy of FMT in restoring a healthy microbiome but also underscore the broader implications of microbiota modulation in enhancing patient health outcomes. This aligns closely with the principles discussed in the context of prebiotics, probiotics, synbiotics, and postbiotics, which similarly aim to optimize gut health by supporting beneficial microbial populations. Just as FMT works by re-establishing a balanced microbiome that can outcompete pathogens, the strategic use of prebiotics and probiotics fosters a diverse gut flora, thereby promoting resilience against dysbiosis and its associated health challenges.

### 3.1. Clostridium Difficile Infection

Clostridium difficile is a spore-forming bacterium that causes a spectrum of gastrointestinal diseases ranging from mild diarrhea to severe colitis [119]. The rise in CDI incidence has been closely linked to antibiotic use, which disrupts the normal gut microbiota, creating an ecological niche for *C. difficile* to thrive [120]. Clinical studies have repeatedly demonstrated the efficacy of FMT in treating recurrent CDI, which often proves resistant to standard antibiotic therapies. A meta-analysis by Kassam et al. (2013) has highlighted that FMT achieves a success rate exceeding 90% in resolving recurrent CDI, with some studies reporting success rates as high as 96% in specific populations, markedly improving patient prognosis and quality of life. This high success rate is attributed to FMT’s ability to rapidly restore microbial diversity, a critical factor for re-establishing a balanced gut ecosystem that can suppress *C. difficile* colonization [121,122,123]. The mechanisms by which FMT exerts its therapeutic effects in CDI involve several key processes. FMT introduces a diverse array of microbiota that competes with *C. difficile* for nutrients and binding sites on the intestinal epithelium [124]. This competition is crucial in limiting the resources available for *C. difficile* proliferation. A study has indicated that, after FMT, the diversity of the gut microbiota can increase by approximately 50%, significantly enhancing the competitive environment against pathogens [125]. Additionally, the restored microbiota can enhance the integrity of the intestinal mucosal barrier, reducing the permeability of the gut lining and preventing the translocation of pathogens and toxins [126]. Furthermore, a healthy microbiota supports the local immune response, producing metabolites, such as short-chain fatty acids (SCFAs), that have anti-inflammatory properties and which promote a more robust immune defense against pathogens [127]. Recent studies have further elucidated the role of gut microbiota-derived compounds in modulating inflammatory responses and promoting gut health [128].

### 3.2. Helicobacter pylori

*Helicobacter pylori* is a gram-negative bacterium implicated in various gastric diseases, including chronic gastritis [129], peptic ulcers [130], and gastric cancer [131]. Standard treatment regimens often involve a combination of antibiotics and proton pump inhibitors; however, these approaches can lead to antibiotic resistance and undesirable side effects. Recent research has explored the potential of FMT as an alternative strategy for *H. pylori* eradication. Studies suggest that FMT can alter the gastric microbiome, potentially enhancing the effectiveness of traditional *H. pylori* treatments. For instance, Xue et al. (2022) have reported that FMT can significantly improve the success rates of *H. pylori* eradication from 50% to 80% by restoring microbial diversity and shifting the gastric microbial community composition in a beneficial direction [132]. Additionally, other studies have demonstrated that microbiota modulation through FMT may inhibit *H. pylori* colonization, thereby improving eradication rates [133,134].

Initial clinical evidence suggests that FMT may lead to successful *H. pylori* eradication in patients who are refractory to standard therapies. The mechanisms by which FMT may achieve this include microbiota modulation, wherein FMT can introduce beneficial bacteria that inhibit *H. pylori* colonization, potentially through competitive exclusion or the production of antimicrobial substances. For example, Bifidobacterium and Lactobacillus species have been shown to significantly inhibit *H. pylori* growth in vitro [135]. Additionally, a restored microbiome may bolster the gastric mucosal barrier and improve immune responses, providing additional protection against *H. pylori* infection. The complex interactions within the gastric microbiome suggest that FMT emerges as a promising tool to combat *H. pylori* infections, particularly in difficult-to-treat cases [135,136].

## 4. Advantages and Disadvantages of Fecal Microbiota Transplantation

FMT represents a significant advancement in treating various gastrointestinal disorders, but it is essential to weigh its advantages against potential drawbacks. One of the foremost advantages of FMT is its remarkable efficacy, particularly in the context of recurrent CDI. The high success rates, often reported to be above 90%, underscore FMT’s effectiveness [121,122,123] when compared with traditional therapies, which frequently yield lower resolution rates (typically around 60% to 70% for standard antibiotic treatments) [94,137]. This efficacy is especially crucial for patients with recurrent infections who have exhausted other treatment options [138]. Furthermore, FMT is unparalleled in its ability to restore the diversity of the gut microbiota, which is essential for optimal gastrointestinal function. The reintroduction of a diverse microbiome is associated with numerous health benefits, including improved digestion, enhanced immune responses, and reduced inflammation [139,140]. This restoration can lead to long-term health improvements, not only in gastrointestinal health but also in metabolic and immune-related conditions. Beyond CDI, FMT shows potential in addressing other conditions linked to dysbiosis, such as inflammatory bowel disease (IBD), metabolic syndrome, and even liver diseases [23,141]. This versatility suggests that FMT could become a cornerstone of therapeutic strategies aimed at restoring microbial balance across various disease states.

Despite the apparent benefits of FMT, there are inherent risks associated with the procedure. Adverse events can include gastrointestinal discomfort, infections, and, in rare cases, severe complications, such as bowel perforation. A comprehensive meta-analysis has documented these risks, highlighting that approximately 2% to 10% of patients may experience mild to moderate adverse events, with severe complications occurring in less than 1% of cases [1,142]. Specifically, 3% of participants in a large cohort study reported transient diarrhea post-FMT [143]. The necessity of stringent donor screening and careful patient selection to mitigate adverse outcomes is crucial [144]. The regulatory landscape surrounding FMT is complex and varies widely across different countries. The lack of standardized protocols for donor screening, microbiota preparation, and administration raises ethical concerns about the safety and efficacy of FMT. Regulatory bodies are still grappling with how to classify FMT, leading to uncertainty in clinical practice and research [145]. Furthermore, while FMT has demonstrated short-term efficacy, the long-term consequences of introducing a foreign microbiome into a recipient’s gut remain largely unknown. Potential risks include the development of new dysbiosis [146], altered metabolic pathways [147], or even the emergence of pathogenic strains [148]. Continuous monitoring and research are vital to understanding the full implications of FMT on long-term health [149].

## 5. Conclusions

In conclusion, prebiotics modulate gut microbiota and enhance their integrity, while probiotics have demonstrated efficacy in various gastrointestinal disorders but also their potential in terms of hypercholesterolemia, improving lipid profiles, and type 2 diabetes mellitus. Synbiotics have synergistic effects in improving gastrointestinal health and immunity. Furthermore, postbiotics act as bioactive compounds, with promising antibacterial and anti-inflammatory properties. Lastly, fecal microbiota transplantation, while not devoid of risks, appears to have a role in restoring damaged microbiota balance. While gastrointestinal disorders have been largely investigated as a logical target for these interventions, future research should also address metabolic diseases, like cardiovascular diseases and hyperuricemia.

## Figures and Tables

**Table 1:** The role of probiotics, prebiotics, synbiotics, and postbiotics on human health.

Functional Food	Sample	Study Design	Dose and Treatment	Duration	Study Outcomes	References
**Probiotic strain and food source**	Hypercholesterolaemic (*n* = 114)	Double-blind RCT	Probiotic capsule: 115 g yoghurt + 10 BSH-active *L. reuteri* NCIMB 30242 (g 5 × 10^9^(CFU/g) twice per day	6 weeks	- LDL-C, TC, non-HDL-C, apoB-100 - TAG and HDL-C	[21]
***Lactobacillus reuteri* NCIMB 30242 (Cardiovivae)** **Yoghurt**						
***Enterococcus faecium* CRL 183 and *Lactobacillus helveticus* 416** **Fermented soy**	Healthy man with (TC) > 5.17 mmol/L and < 6.21 mmol/L (*n* = 49)	Double-blind RCT	Group SP (*n* = 17), 200 mL of the fermented soy product, containing (SP-10^10^ CFU) daily Group ISP (*n* = 17), 50 mg of total isoflavones/100 g of the fermented soy product with isoflavones daily	42 days	- Reduces risk of CVD - Antioxidant properties - Anti-inflammatory action	[22]
***Lactobacillus plantarum* CJLP243** **Kimchi**	Patients with rectal cancer undergoing ileostomy reversal	Double-blind RCT	Probiotic treatment once per day preoperative	21 days	- No significant evidence of restoring bowel function - Improvements in some subscale bowel function measures	[23,24]
***Lactobacillus plantarum* K50** **Kimchi**	Healthy adults, BMI (25–30 kg/m^2^)	Double-blind RCT	LPK isolated from kimchi	12 weeks	- Body weight, fat mass, abdominal fat - Cholesterol and triglyceride - Changes in gut microbiota: *L. plantarum* ↑, Actinobacteria - Changes in visceral adiposity	[25]
***Bacillus coagulans*** **Whey protein powder**	Resistance-trained males	Double-blind RCT	Treatment 1: 20 g whey protein powder + *Bacillus coagulans* Unique IS-2, once per day	2 months	- Protein absorption - Branched chain amino acids (BCAA) - Isoleucine, leucine, valine - Lower body muscle power strength	[26]
**Prebiotic strain and food source**	Overweight adults predisposed to metabolic syndrome (*n* = 45)	Double-blind RCT	5.5 g of Bi2 muno and B-GOS	12 weeks	- Enhanced immune response - TC, TG, and TC HDL (CPR), insulin	[27]
**Trans-galactooligosaccharide mixture [Bi2 muno (B-GOS)]**						
**MSPrebiotic^®^**	Elderly and middle-aged Canadians (*n* = 84)	Double-blind RCT	MSPrebiotic^®^	3 months	- *Bifidobacterium* in both elderly and middle-aged populations - SCFA (elderly) - *Proteobacteria* (elderly)	[28]
**Bimuno^®^ galactooligosaccharide (B-GOS^®^)**	Autistic children (*n* = 30)	Double-blind RCT	B-GOS^®^ mixture (Bimuno^®^; 1.8 g: 80% GOS content)	6 weeks	- Improvement in gut microbiota composition: *Bifidobacterium* spp. and *Veillonellaceae* family *Lachnospiraceae* family *Faecalibacterium prausnitzii* and *Bacteroides* spp. - Lower abdominal pain and bowel movement *-* Improvements in anti-social behaviors - Significant changes in fecal and urine metabolites	[29]
**Orafti^®^ inulin-type fructans**	Healthy children (*n* = 209)	Double-blind RCT	6 g/day prebiotic inulin-type fructans	24 weeks	- Induced positive changes in the composition of the gut microbiota - Enhanced immune-modulating effects of prebiotics - Inhibited antibiotic-induced disturbances	[30]
**Inulin and resistant starch**	Lean and overweight/obese adults (*n* = 26) with (BMI) ≥20 and ≤35 kg/m^2^	Double-blind RCT	75 g GLU or 75 g GLU + 24 g oliggo-fiber instant IN or 75 g GLU + 28 g RS dissolved in 300 mL water		- No effect on GLP-1 or PYY responses - Reduction in ghrelin	[31]
**Postbiotic strain and food source**	In vivo mice	Double-blind RCT	Bacterial pellets 10^9^ CFU Aerobic incubation at 37 °C Lactobacillus rhamnoses 1 × 10^9^ CFU or *Escherichia* coli 1 × 10^8^ CFU at 37 °C in 5% CO_2_	24–48 h 3 h	*Escherichia* coli’s overall activity and infectivity is decreased, which stops the intestinal inflammation of the mice.	[32]
**Nutraceuticals products** ***Lactobacillus rhamnosus***	In vivo *n* = 12 post weaning lambs	Double-blind RCT	Washing the active cultures with 0.85%NaCl adjusted to 10^9^ CFU/mL. Then, 10%, incubated at 30 °C, collection of supernatants by centrifugation, at 10,000× *g* at 4 °C	10 h 15 min	Dietary postbiotics decreased serum lipid peroxidation, increased hepatic antioxidant enzyme activity, improved ruminal barrier integrity, and increased antioxidant activity.	[33]
**Postbiotic of *Lactobacillus plantarum***						
**Postbiotic of *L. bulgaricus* and *S. thermophilus***	In vivo mouse model	Double-blind RCT	*S. thermophilus* and *L. bulgaricus* cultured at 37 °C the growth about 5 × 10^8^ CFU and 1 × 10^9^ CFU	15 min	Effective at slowing the progression of colitis in mice	[34]
**Exopolysaccharide-producing strain *S. thermophilus***	In vitro Food: Cheddar cheese	Double-blind RCT	Physicochemical analysis of CRMP Three replicate cheese-making trials Samples: - Direct-acidified cheese - Cheese with *Streptococcus thermophilus* TM11 24.8 ± 0.4 - Cheese with *Streptococcus thermophilus* SP1.1 23.5 ± 0.3	45 days	Increased moisture content and high yield Improved cheese yield and texture Improved product performance	[18]
**Supernatant of *L. sakei***	In vitro Food: Grilled beef	Double-blind RCT	10^3^ CFU/g *E. coli* or *Listeria monocytogenes*	120 h	Decreased *E. coli* and *Listeria monocytogenes*	[35]
**Synbiotic strain and food source**	In vivo *n* = 115 pregnant women	Double-blind RCT	Two groups to receive a daily synbiotic capsule 500 mg of *L. acidophilus* 5 × 10^10^ CFU/g *L. plantarum* 1.5 × 10^10^ CFU/g *L*. fermentum 7 × 10^9^ CFU/g, L. Gasseri (2 × 10^10^ CFU/g), 38.5 mg of fructo-oligo-saccharides or placebo	6 weeks	A significant decrease in logTG/HDL-C ratio with a medium–low effect size	[36]
**Synbiotic capsule** ***Lactobacillus acidophilus*** ***Lactobacillus plantarum*** ***Lactobacillus fermentum, Lactobacillus Gasseri* and fructo-oligo-saccharides**						
***L. acidophilus*** **+ cinnamon powder**	In vivo *n* = 136 T2DM patients	Double-blind RCT	G1: *Lactobacillus acidophilus* 10^8^ CFU and 0.5 g of cinnamon powder (synbiotic) G2: Probiotic *Lactobacillus acidophilus* G3: cinnamon powder G4: a placebo	3 months	Increase of antioxidant enzymes	[37]
**Synbiotic capsule containing *L. acidophilus*, *L. casei*, and *B. bifidum* + inulin**	In vivo *n* = 60 diabetic HD patients	Double-blind RCT	(2 × 10^9^ CFU/g each), plus 0.8 g/day of inulin (*n* = 30) or placebo (*n* = 30)	12 weeks	A considerable boost in overall antioxidant capacity and favorable benefits on glycemic management, inflammatory biomarkers, and oxidative stress in diabetic patients receiving HD	[38]
***S. cerevisiae*** **+ Mannanoligosaccharides (MOS)**	In vivo *n* = 100 chicks	Double-blind RCT	1. Control base diet 2. Base diet + mannan-oligosaccharide 2 g/kg + 0.5 g/kg of grower diets 3. Base diet + probiotic 3 g/kg diet, saccharomyces cerevisiae 4. Base diet + mixture of pre- and probiotics-synbiotic	6 weeks	Weight gain, less *E. coli* in SI and cecum	[39]
***S. cerevisiae*** **+ Inulin**	In vivo *n* = 15 calves	Double-blind RCT	Synbiotic inulin 6 g *+ S. cerevisiae* strain 1026, 5 g	56 days	Positively influenced by increased pH in the rumen, abomasum, and intestines.	[40]
**Bifidobacterium, *Lactobacillus*, and *S. thermophilus* + fructo-oligosaccharide**	In vivo *n* = 70	Double-blind RCT	500 mg/day	9 weeks	Improved HbA1c, BMI, and microalbuminuria.	[41]
***Bifidobacterium lactis*** **+ fructo-oligosaccharides**	In vivo *n* = 27	Double-blind RCT	5 × 10^9^ CFU/bag + 4.95 g/bag	30 days	Enhanced intestinal performance Lowered levels of IL-6, IL-8, IL-17, and interferon-gamma (IFNγ)	[42]

**Table 2:** Category-wise key benefits from the summary of Table 1.

Category	Definition	Health Role	Key Benefits	Mechanisms of Action	Sources
Prebiotics	Non-digestible food ingredients that promote beneficial gut bacteria	Enhance gut health, improve digestion, and reduce inflammation	Prebiotics show improved immune function, balanced gut microbiota, increased lactobacilli and bifidobacteria, and decreased infections.	Promote the growth of beneficial bacteria and alter gut microbiota composition.	Chicory root, garlic, onions, bananas
Probiotics	Live microorganisms that confer health benefits when consumed in adequate amounts	Improve gut microbiota, enhance immunity, prevent diarrhea	Significant improvement in fasting blood sugar levels from 177.3 ± 23.02 mg/dL to 147.70 ± 3.71 mg/dL in T2DM patients.	Enhance gut barrier function, produce antimicrobial substances, modulate immune responses.	Yogurt, kefir, sauerkraut
Postbiotics	Metabolic byproducts of probiotic bacteria	Modulate gut health, improve immune response, and provide antioxidant effects	*E. coli* count in the small intestine decreased from 9.79 log CFU/g (control) to 7.49 log CFU/g (synbiotic group).	Influence gut microbiota composition, reduce intestinal inflammation, enhance antioxidant enzyme activity.	Fermented foods, dairy products
Synbiotics	Combination of probiotics and prebiotics	Support and enhance the survival of probiotics, improve gut health	Average body weight in broilers was 2299.19 g for the synbiotic group compared with 2058.1 g for the placebo group.	Improve gut microbiota diversity, enhance the absorption of nutrients, synergistic effects between probiotics and prebiotics.	Supplements, functional foods

## Data Availability

No new data were created or analyzed in this study.
